# Psychological Assessment of Family Caregivers of Patients With COVID-19 in the United States of America and India

**DOI:** 10.7759/cureus.29267

**Published:** 2022-09-17

**Authors:** Yashendra Sethi, Oroshay Kaiwan, Mahmoud Bassiony, Vidhi Vora, Pratik Agarwal, Neeraj Gajwani, Keshav Garg, B Siva Sai, Adyut Prakash, Snehal Gohel, Debabrata Roy

**Affiliations:** 1 Department of Medicine, Government Doon Medical College, Dehradun, IND; 2 Medicine, Northeast Ohio Medical University, Rootstown, USA; 3 Faculty of Medicine, Alexandria University, Alexandria, EGY; 4 Department of Medicine, Lokmanya Tilak Municipal Medical College, Mumbai, IND; 5 Department of Medicine, AMA School of Medicine, Manila, PHL; 6 Department of Medicine, Government Medical College & Hospital, Chandigarh, IND; 7 Department of Medicine, Government Medical College, Anantapur, IND; 8 Department of Medicine, Calcutta National Medical College and Hospital, Kolkata, IND; 9 Obstetrics and Gynaecology, GMERS Medical College, Sola, IND; 10 Department of Community Medicine, Government Doon Medical College, Dehradun, IND

**Keywords:** family caregivers, families of covid patients, sleep disturbances, anxiety, stress, covid

## Abstract

Background and objective

The coronavirus disease 2019 (COVID-19) pandemic has highlighted the shortcomings worldwide in terms of preparedness protocols related to epidemics. A key area of research that is evidently overlooked across the globe is the mental health of family caregivers taking care of patients with COVID-19. In light of this, this study aimed to engage in a comparative analysis between the two worst affected countries, India and the United States of America (USA), which differ considerably in their demography, socio-epidemiological factors, and health system efficiency.

Methods

A cross-sectional study was conducted among 1,250 family caregivers of patients with COVID-19 in India and the USA to assess their stress, anxiety, and sleep disturbance levels using the 10-item Perceived Stress Scale (PSS-10), the 7-item Generalized Anxiety Disorder (GAD-7) scale, and the Pittsburgh Sleep Quality Index (PSQI), respectively. Psychological assessment questionnaires were administered through online mode, which gathered demographic information and responses on several self-reporting scales. The main outcome measures were self-reported ratings on PSS, GAD-7 scale, and PSQI.

Results

We found that 75.4% of the family members of COVID-19 patients suffered from mental health issues. The scores of all three scales were higher in caregivers from the USA than in India, more evident and pronounced in caregivers of hospitalized patients. The test scores were statistically significant (p<0.05) indicating a negative impact of having a dependent member in the family, being married, being of younger age, and having a longer duration of COVID-19 infection. Vaccines were found to have a life-enhancing effect.

Conclusion

Our findings highlight that the mental health of family caregivers is an ignored aspect and must be addressed. We recommend the implementation of well-researched and appropriate legislation, treatment programs, and health policies that involve not only the patients but also their families.

## Introduction

The coronavirus disease 2019 (COVID-19) emerged as a pandemic in 2019 and was declared a public health emergency of international concern (PHEIC) by the WHO on January 30, 2020 [1]. The United States of America (USA) and India have been the most affected countries, reporting a total of 97 million and 44 million cases respectively, amounting to approximately 25% of the world’s total cases [2]. Pandemic-induced market instability and the instituted lockdowns further exacerbated this situation by increasing unemployment from 6.7% on March 15, 2021, to 26% on April 19, 2021, in India. During the initial phase of the lockdown, India approximately lost over 4.2 billion USD per day [3]. On the other hand, the gross domestic product (GDP) of the USA contracted by 3.5%, representing the first major contraction since the 2008 financial crisis [4].

In addition to its economic impact, COVID-19 has also affected people's short- and long-term health, including their mental health [5]. The psychological effect of the pandemic has manifested as depression, anxiety, and stress in COVID-19 patients, healthcare workers, and family members of patients [6,7]. A study conducted in the USA identified the prevalence of anxiety, depression, and psychological distress to be 42%, 39%, and 39% respectively [8]. "Health anxiety" rises as a product of either misinformation, lack of health education, or uncertainty associated with the pandemic. At an individual level, this can translate into maladaptive behaviors including repeated medical consultations, healthcare avoidance despite illness, and hoarding of items. At a broader societal level, it can often lead to mistrust of public authorities and scapegoating of particular population groups [9].

Understandably, COVID-19 infection in any family member results in psychological trauma due to social isolation, increased economic stress, and stigmatization by society. Therefore, it is important to acknowledge and understand the role that caregivers have played in this pandemic and its subsequent effects on their mental health. Studies have shown that informal caregivers of patients who were discharged from hospitals can be classified into two categories: (1) caregivers who are motivated by altruism and have a positive impact on the patient and their health and (2) those who provide care based on familial norms. Both types of caregivers, especially those directly involved in the care of patients, are pushed toward physical and mental burnout and hence experience depression, increased anxiety, and deteriorating physical health. Unsatisfactory supportive care and lack of health information are the major contributors to anxiety, stress, and sleep disturbances among caregivers [10].

The economic and social impact of COVID-19 has been the greatest on India and the USA. The USA, being a developed country, has a stronger healthcare infrastructure with a doctor-to-population ratio of 2.6/1,000, whereas India, an emerging and developing country (EDC) with a doctor-to-population ratio of 0.9/1000, has a healthcare system that is encumbered [11]. Also, the USA has a higher proportion of people who received at least one dose of the COVID-19 vaccine (77.75%) than India (72.15%) [12]. Despite these factors, the deaths due to COVID-19 in the USA amount to 3,062 per million, whereas the number is 373 per million in India. These differences can be attributed to socio-cultural differences between the populations of the two countries. India has a younger population with clinically favorable genetic polymorphism in COVID-19 receptors. There was more stringent enforcement of lockdowns in India compared to the USA. Furthermore, there is an increased prevalence of obesity in the USA than in India [13]. On the other hand, due to a large proportion of the population being socially and economically vulnerable, a high burden of pre-existing undiagnosed mental illnesses, and limited mental health services, the stress, anxiety, and sleep disturbances in the setting of COVID-19 in India are more challenging to deal with [14-16].

Although recent studies have looked into the impact of the COVID-19 pandemic on mental health, they have largely focused on the suffering of the patients and very few have focused on the family members and caregivers of the patients. To date, there has been no study highlighting the prevalence of symptoms of stress, anxiety, and disturbances in sleep quality among the family members of patients with COVID-19 while also comparing the variations based on demographic, environmental, physical, social, and economic factors. Also, there have been no attempts to compare the aforementioned deleterious effects on a developed (USA) versus an emerging and developing nation (India). Our study aims to address this gap in the literature by exploring the effects of the pandemic on the mental health of family caregivers of COVID-19 patients in India and the USA.

## Materials and methods

Study design and setting

A community-based cross-sectional study was conducted to estimate the prevalence of stress, anxiety, and sleep disturbances among family members of patients with COVID-19. The study was conducted among family members of patients who suffered from COVID-19 in India and the USA, the two countries worst affected by the pandemic. We employed purposive sampling to distribute the questionnaire over WhatsApp and email to all the participants, covering rural as well as urban areas.

Participant selection

Participation in the study was voluntary and anonymous. Participants were selected from both countries as per well-defined inclusion and exclusion criteria.

Inclusion and exclusion criteria

We included family members or informal caregivers who were either directly or indirectly involved in the care of the patient. We excluded the respondents who had a history of pre-existing mental illness, as these illnesses can act as confounders that can alter self-reports and misrepresent the effect of COVID on the mental health of caregivers. 

Data collection and study instruments

Psychological assessment questionnaires were administered to all the subjects. The study objectives and the risks and benefits of participation were clearly explained, and detailed informed consent was taken online before providing the questionnaire. The main outcome measures were self-reported ratings on stress, anxiety, and sleep quality. Data were collected from 1,272 participants from India and the USA, with data collection stopped upon reaching a sample of 625 from each country. Among the total participants who filled out the form, 98.3% (n=1,250) gave consent and filled the form further while 1.7% (n=22) denied it and landed on the "thank you" page directly. The questionnaire, administered via Google Form, had 12 sections including consent, basic demographic details, possible confounders, means of handling stress, and psychosocial assessment scales, i.e., scales for stress, anxiety, and sleep. The language of the survey was confined to English, to ensure uniformity and the validity of the scales used.

The psychological responses from family caregivers were assessed using three established and globally validated self-reporting psychological instruments/scales:

The 10-item Perceived Stress Scale (PSS-10)

It is a 10-item self-reported scale used to measure global perceived stress during the preceding month. Scores range from 0 to 40 with higher scores indicating a higher risk for future distress [17].

The 7-item Generalized Anxiety Disorder (GAD-7) scale 

It is a 7-item self-administered scale used as a screening measure of anxiety. The scores of 5, 10, and 15 are taken as indicators of mild, moderate, and severe anxiety, respectively [18].

The Pittsburgh Sleep Quality Index (PSQI)

It determines sleep quality in the previous month, using seven assessment indicators. Scores per item range from 0 to 3 points, and total scores range from 0 to 21 points. A total PSQI score <5 indicates good sleep quality, while a score ≥5 indicates poor sleep quality [19].

Study size and sampling methodology

A purposive sampling model was used for data collection with the questionnaire distributed online to people in various parts of the two countries, allowing for the inclusion of a comprehensive representative sample. The sample size of approximately 1,250 (625 from each country) was calculated using the G*Power statistical analysis software using the following parameters: effect size: 0.2, alpha error probability: 0.03, power (1-beta): 0.95, and allocation ratio N2/N1: 1.

Statistical analysis

The collected data were analyzed primarily based on scores of scales, and individual responses to questions of self-reporting scales were also analyzed qualitatively to gain a deeper insight. Quantitative variables were analyzed using SPSS Statistics (IBM Corp., Armonk, NY) version 27.0 for Windows. All comparative analyses were done using standard statistical tools by applying parametric and nonparametric tests comparing all variables and confounders. The data were first qualitatively assessed using demographic analysis and normality assumptions (Shapiro-Wilk test); qualitative variables were analyzed using diagrammatic charts and quantitative data were then analyzed using the Student's t-test and the analysis of variance (ANOVA) test contrasting the scores of the implicated scales.

Ethical approval and consent

The study was approved by the Ethics Committee of the Faculty of Medicine - Alexandria University with IRB no. 00012098 and FWA no. 00018699, and all participants provided online written informed consent.

## Results

The study involved 1,250 participants, 625 from each country. Demographic information was collected to account for its impact on stress, anxiety, and disturbances in sleep and to eliminate any confounding variables such as gender, age, income, and place of residence that can impact a person’s health. Of the 1,250 participants, 55% were male (689) and 44.9% were female (561). Most participants were young adults, aged 18-29 years (53.4%) and were either graduates (49.2%) or undergraduates (24.2%). Economically, most people reported belonging to the middle class (77.7%), with fewer in the lower (10.1%) and upper classes (12.2%). Most participants lived in urban areas (79%), with fewer in rural (11.7%) and suburban (9.3%) areas. This study found that 75.4% of caregivers of COVID-19 patients suffered from some form of mental health distress.

Stress

**Figure 1:**
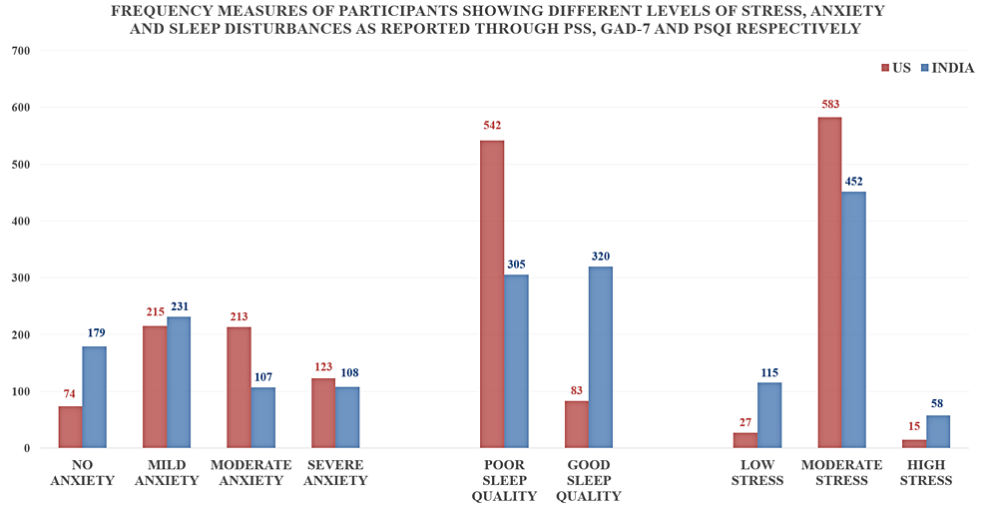
Frequency measures of participants showing different levels of stress, anxiety, and sleep disturbances as reported through PSS-10, GAD-7, and PSQI respectively Anxiety levels among participants were found to be higher among respondents from the US. Also, disturbances in sleep quality were seen to be more prevalent in respondents from the US. Overall higher prevalence of stress was reported more in the US, but more entries of high stress were seen in respondents from India, which may be attributed to differences in the perception of stress influenced by a variety of socio-epidemiological factors PSS: Perceived Stress Scale; GAD-7: 7-item Generalized Anxiety Disorder scale; PSQI: Pittsburgh Sleep Quality Index

Moderate to high stress was found in 95.68% of the study population from the USA and 81.60% of the Indian subjects (Figure 1). It was more severely associated with the female gender (p<0.001), those taking care of severe/hospitalized patients (p<0.001), those with underlying chronic diseases (p=0.010), respondent being the primary source of income in the family (p=0.003), and longer duration of COVID-19 infection (p<0.001) (Tables 1, 2).

**Table 1:** Factors associated with anxiety, sleep quality, and stress levels *Statistically significant Mean scores of GAD-7, PSS, and PSQI compared across various confounding variable groups using the Student's t-test PSS: Perceived Stress Scale; GAD-7: 7-item Generalized Anxiety Disorder scale; PSQI: Pittsburgh Sleep Quality Index

Variables			Anxiety		Sleep quality		Stress levels	
		N (%)	Mean	P-value	Mean	P-value	Mean	P-value
Gender	Male	689 (55.1%)	9.042	0.008*	7.909	0.465	18.446	<0.001*
	Female	561 (44.9%)	9.982		7.736		19.856	
Country	India	625 (50.0%)	8.506	<0.001*	6.110	<0.001*	18.438	<0.001*
	United States	625 (50.0%)	10.422		9.552		19.718	
Direct involvement in providing care to the patient	Yes	922 (73.7%)	9.974	<0.001*	8.226	<0.001*	19.206	0.150
	No	328 (26.3%)	8.030		6.723		18.720	
Caring for home-isolated or hospitalized patient	Home-isolated	661 (52.9%)	8.286	<0.001*	7.100	<0.001*	18.212	<0.001*
	Hospitalized	589 (47.1%)	10.786		8.652		20.051	
Deaths in neighborhood/of relatives due to severe COVID-19 infection	Yes	893 (71.4%)	10.063	<0.001*	8.083	0.001*	19.559	<0.001*
	No	357 (28.6%)	7.966		7.202		17.877	
Respondent is the primary source of income in the family	Yes	539 (43.1%)	10.727	<0.001*	9.553	<0.001*	19.586	0.003*
	No	711 (56.9%)	8.506		6.526		18.693	
Patient is the primary source of income in the family	Yes	569 (45.5%)	10.028	0.004*	8.480	<0.001*	19.510	0.008*
	No	681 (54.5%)	8.993		7.289		18.718	
Family size	4 or below	731 (58.5%)	9.631	0.265	7.938	0.278	19.150	0.565
	5 and above	519 (41.5%)	9.229		7.600		18.977	
Dependent members in the family	Yes	856 (68.5%)	9.950	<0.001*	8.049	0.006*	19.192	0.262
	No	394 (31.5%)	8.409		7.358		18.83	
Known history of chronic diseases	Yes	252 (20.1%)	10.175	0.044*	9.492	<0.001*	19.837	0.010*
	No	998 (79.8%)	9.285		7.412		18.887	

**Table 2:** Stress, anxiety, sleep quality, and associated factors *Statistically significant Variables reported in the questionnaire: marital status: single/married/divorced/widowed; residence: urban, rural, suburban; age: 18-29/30-44/45-59/60-85/>85 years; income group: lower/middle/upper; year of COVID-19 infection: 2019/2020/2021; duration of COVID-19 infection: <10/10-20/>20 days; patient outcome: still infected/recovered/deceased; impact of news: positive/negative/no impact; respondent’s vaccination status: fully/partially/not vaccinated; patient’s vaccination status: fully/partially/not vaccinated PSS: Perceived Stress Scale; GAD-7: 7-item Generalized Anxiety Disorder scale; PSQI: Pittsburgh Sleep Quality Index

Variables	Anxiety (GAD-7)		Stress (PSS)		Sleep quality (PSQI)	
	F	P-value	F	P-value	F	P-value
Marital status	7.270	<0.001*	1.425	0.223	18.041	<0.001*
Area of residence	5.008	0.007*	0.561	0.571	2.173	0.114
Age	4.297	0.005*	3.499	0.015*	21.991	<0.001*
Income group	0.859	0.424	11.872	<0.001*	10.646	<0.001*
Year of COVID-19 infection	4.499	0.011*	1.048	0.351	21.561	<0.001*
Duration of COVID-19 infection	40.727	<0.001*	27.589	<0.001*	50.76	<0.001*
Patient outcome	0.603	0.547	16.979	<0.001*	10.873	<0.001*
Impact of news sources on mental health	43.182	<0.001*	41.603	<0.001*	38.415	<0.001*
Respondent’s COVID-19 vaccination status	16.419	<0.001*	1.350	0.260	6.909	0.001*
Patient’s COVID-19 vaccination status	5.032	0.007*	0.302	0.739	11.522	<0.001*

Anxiety

A higher prevalence of moderate (34.08%) and severe anxiety (19.68%) levels were observed among respondents in the USA, whereas mild anxiety levels (36.96%) were more prevalent in India (Figure 1). Certain risk factors were identified; females were affected more than males (p=0.008). Also, those who had any severe case or death in their close environment (p<0.001) had a higher incidence of anxiety (Table 1). The anxiety levels were exacerbated by the hospitalization of the patient (p<0.001), the presence of a dependent member in the family (p<0.001), being married (p<0.001), younger age (p=0.005), and longer duration (>20 days) of COVID-19 infection (p<0.001) (Table 2).

Sleep

Sleep disturbances were more prevalent in the USA (p<0.001) with 86.72% reporting poor sleep quality while only 48.8% reported poor sleep quality in India (Figure 1). Disturbed sleep was considered by most as a symptom that required consultation, followed by irritability, general feeling of dislike, hearing their own heart sounds, and not feeling motivated. Sleep quality was assessed to be poor in those directly involved in providing care to the patients (p<0.001); poor sleep quality was also associated with caring for patients with comorbidities (p<0.001) or hospitalized patients (p<0.001), the presence of a dependent member in the family (p=0.006), being the primary source of income in the family (p<0.001), and longer duration of COVID-19 infection (p<0.001) (Tables 1, 2).

The vaccination status of respondents also played a statistically significant role in determining the levels of anxiety (p<0.001) and sleep disturbances (p=0.001) with vaccinated individuals scoring lower on these self-reporting scales. Similarly, the levels of anxiety (p=0.007) and sleep disturbances (p<0.001) were found to be lower in groups with vaccinated patients (Table 2).

**Figure 2:**
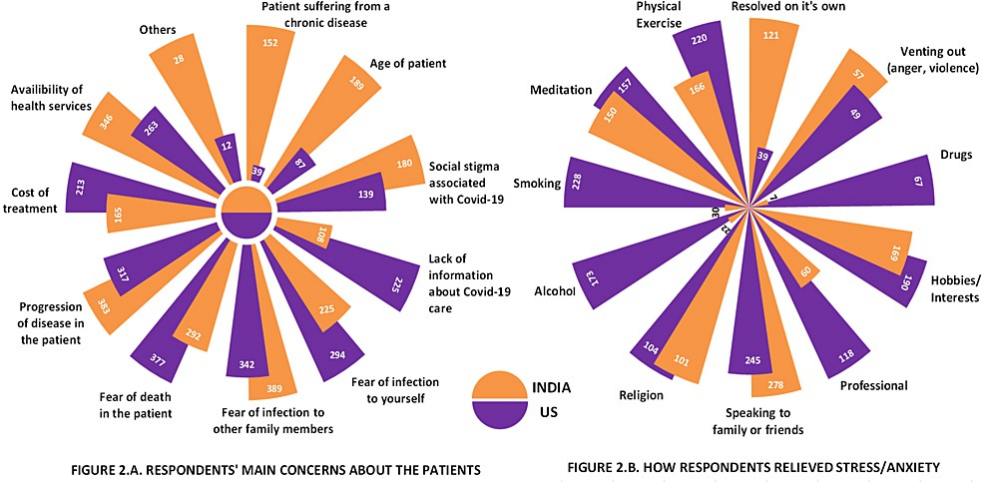
The main concerns of respondents and their methods to relieve stress/anxiety 2A: respondents' main concerns about the patients – the most common concern among respondents from the US was fear of death of a patient while that for respondents from India was fear of infection to family members. 2B: how respondents relieved stress/anxiety – “speaking to family or friends” was reportedly the most common method adopted to relieve stress/anxiety

The main concerns expressed by family members were fear of infection in other family members (58.5%), progression of disease in the patient (56%), fear of the death of the patient (53.5%), and the availability of health services (48.7%). The most common concern among family members of the Indian population was fear of infection to other family members (62.24%) while that from the USA was fear of death of patients (60.32%) (Figure 2A).

In these challenging times, people have adopted various methods to cope with anxiety, stress, and sleep disturbances. About 20% of the respondents have chosen conversation with family members as their main coping mechanism (Figure 2B). Other important stress relievers, especially among USA respondents, included physical exercise (35.2%), hobbies (30.4%), and some unhealthy habits such as smoking (36.48%) or alcohol consumption (27.68%). Conversely, only a small proportion of our respondents sought professional help (14.24%).

To summarize the results, we found that men were more prone to anxiety and sleep disturbances; however, women were more affected by stress. The US subjects exhibited much greater levels of anxiety, stress, and sleep disturbances. Respondents who were directly involved in the care of patients had considerably higher levels of anxiety, sleep disturbances, and stress. Similarly, fatalities in the neighborhood or in the family were associated with higher levels of anxiety. Anxiety, sleep disturbances, and stress levels were higher in sole earners, small families, families with dependent members, and patients with a history of chronic disease (Table 1).

Various factors had an effect on GAD-7, PSS, and PSQI scores among families of COVID-19 patients. These included being married, younger age, and prolonged duration of infection, which were among the leading factors for anxiety, stress, and sleep disturbances. Profound stress and sleep disturbances were found among respondents from lower-income groups. News sources and social media were shown to have a negative impact on mental health leading to anxiety, sleep disturbances, and stress. The vaccination status of respondents also had a statistically significant impact on anxiety and sleep disturbance levels, with vaccinated people scoring lower on these self-reporting scales. Anxiety and sleep disruptions were also shown to be lower in groups caring for vaccinated patients (Table 2).

## Discussion

COVID-19 has highlighted several gaps in the healthcare systems globally, including poor emergency response protocols. Several studies have shown that pandemics result in adverse psychological outcomes among the public [20,21]. This research was conducted to analyze the impact of COVID-19 on the mental health of caregivers of patients from the USA and India.

Our study found that among family members of COVID-19 patients, 44% had moderate to severe anxiety, 88.6% had moderate to severe stress, and around 67% of respondents had poor sleep quality; these findings are in line with the study by Khubchandani et al., whose findings revealed a general prevalence of anxiety (42%) and psychological distress (39%) during the pandemic. The study also indicated that the rate of serious mental health issues such as anxiety and stress has more than doubled during the pandemic [8]. Abrupt changes in the daily routine can cause imbalances in the circadian cycle leading to the development of sleep disorders, which could explain disturbances in sleep quality [22].

India and the USA

Psychological health varies across regions and countries, especially during a massive disease outbreak owing to differences in outbreak severity, the strength of the national economy, government preparedness, and availability and accessibility of medical facilities and health information. Furthermore, the evolving nature of the outbreak and the legislative response in each region also affect the psychological state of the people, especially those involved in patient care [23]. A better healthcare system, the first-world status of the country, and a better economy are expected to have a better impact on the state of mental health, but interestingly, our study suggested the opposite with respondents from the USA reporting higher levels of anxiety, stress, and disturbances in sleep quality (Figure 1, Table 1). This may be attributed to differences between both countries in terms of the general perception of challenges regarding healthcare, the felt needs, and different cultures and coping mechanisms (Figure 2B).

Being a family caregiver during COVID-19

COVID-19, in general, has been seen to have adverse effects on the mental health of the population [6,7], and the caregivers of patients with life-threatening disease have been seen to experience a heavy toll on mental health [10]. In our study, we observed that the respondents who were directly involved in the care of patients had higher scores on the scale assessing anxiety and they also had poor sleep quality; this data, when compared with those not directly involved in patient care, was statistically significant (p<0.001) (Table 1). This connotes that not only being involved in patient care, but the extent of involvement also had a substantial influence on mental health.

Factors affecting mental health

We also explored the possible factors affecting mental health and sleep. Higher stress, anxiety, and sleep disturbances were directly influenced by the patient's health condition as suggested by the presence of a comorbid chronic illness, longer duration of COVID-19 illness, and disease severity (home-isolated vs. hospitalized). This is because the patient being hospitalized means worsening disease severity, which causes greater trauma among the caregivers. This finding aligns with the study conducted by Paul et al., which concluded that the relatives (of patients in ICU) experienced negative physical and psychological impacts due to unidentified needs and this impact might transgress far beyond the acute phase of the illness [24]. Moreover, a longer duration of a patient’s illness leads to prolonged isolation and loneliness experienced by the family, negatively impacting their anxiety, stress, and sleep quality [25,26]. Anxiety was more prevalent among females (Table 1), which is in agreement with previous studies that stated that females have a higher risk of developing anxiety [27]. It was also seen that young individuals were more prone to anxiety, stress, and sleep disturbances (Table 1), which aligned with the findings of Hyun et al., who also suggested that young adults experience high levels of PTSD symptoms and sleep problems [28]. This is also in line with the findings of Cao et al., who stated that young adults, including students, experienced more emotional distress due to school closures, cancelation of social events, lower study efficiency with remote online courses, and postponements of exams. The adults, on the other hand, being in the role of caregivers, faced a different kind of toll [29].

Higher stress, anxiety, and sleep disturbances were also related to some external factors; anxiety and sleep quality were worse in married patients while stress and poor sleep quality were more prevalent in economically backward groups. Other factors included deaths in the neighborhood, news and media, family education, and socioeconomic status (Table 2). These findings are in agreement with various previous studies [30-34]. The vaccination status of both the patient and the respondent positively affected the caregiver’s anxiety and sleep quality. This finding was also seen in research findings by Babicki et al., who found that fully vaccinated individuals had lower levels of anxiety than those waiting to get vaccinated [35].

Limitations 

The study's limitations essentially pertain to the self-reported data collected via electronic mode; an offline mode of data collection through clinician-administered scales can often show differences in results. Another limitation relates to its cross-sectional study design, which lacks the ability to demonstrate a temporal association between exposure and outcome, which could be better portrayed by having a matched control group as in a case-control study design. Furthermore, the questionnaire was administered only in the English language, which might have limited the number and type of participants. Lastly, purposive sampling was used in the study due to the lack of access to national data on COVID-19 patients and their family members.

Future prospects

We hope to pave the way for future researchers to further explore the effect of pandemics on the mental health of caregivers. More studies exploring the effects of cultural, ethnic, regional, economic, and environmental differences need to be conducted. Governments can initiate need-based interventions for vulnerable groups, and provide telepsychiatry consultations and emergency assistance for psychological and behavioral issues. Hopefully, after the identification of these needs, various solutions will be crafted, especially in the domain of public health legislation and policies to address mental health.

## Conclusions

The importance of the mental health of family members of COVID-19 patients cannot be overstated. This research highlights the magnitude of the problem and provides a comparative analysis of the impact of the pandemic between a developed country and an emerging and developing nation. Although measures are being put in place to assess the psychological status and prevent sleep and mental disorders, more work needs to be done to ensure adequate response to increasing cases of anxiety, stress, and poor sleep quality. The indicative survey revealed the association of the issue with factors such as the severity of the disease, isolation, gender, healthcare systems or lack thereof, and the role of mass media, all of which critically influence the psychological and physiological well-being of these family members, and could be a compass for the development of intervention programs to resolve such issues. These issues can also be effectively mitigated by proper health promotion and public health education. Emphasis should be placed on improving healthcare systems and equipping hospitals with sufficient resources to manage future pandemics. Well-researched and appropriate legislation, treatment programs, and health policies should be implemented to integrate healthcare services and introduce psychological care at the earliest and at all levels.
